# Regional vulnerability for COVID-19 in Cameroon

**DOI:** 10.11604/pamj.supp.2020.37.1.26167

**Published:** 2020-09-23

**Authors:** Seth David Judson, Kevin Yana Njabo, Judith Ndongo Torimiro

**Affiliations:** 1 Department of Medicine, University of Washington, Seattle, USA,; 2 Center for Tropical Research, University of California, Los Angeles, USA,; 3 The Chantal Biya International Reference Centre for Research on the Prevention and Management of HIV/AIDS (CIRCB), Yaoundé, Cameroon

**Keywords:** COVID-19, SARS-CoV-2, coronavirus, risk factors, co-morbidities, Cameroon, Africa, epidemiology, vulnerability, transmission

## Abstract

**Introduction:**

few studies have assessed risk for coronavirus disease 2019 (COVID-19) within African countries. Here we examine differences in vulnerability to COVID-19 among the ten administrative regions and two major cities of Cameroon based on epidemiological risk factors and access to healthcare resources.

**Methods:**

regional epidemiological and healthcare access vulnerability indices were created and compared with cumulative COVID-19 cases, case fatality rates, co-morbidities, and healthcare resources in Cameroon.

**Results:**

based on epidemiological risk factors, populations in the East Region, Douala (in the Littoral Region), West Region, and Yaoundé (in the Center Region) are at highest risk for COVID-19. Meanwhile, the North, Far North, East, and Adamawa Regions had the most healthcare access vulnerability. COVID-19 cases per population were highest in the Center, Littoral, and East Regions. Case fatality rates were greatest in the North Region. Potential co-morbidities with greater prevalence among COVID-19 patients included male sex, hypertension, and diabetes.

**Conclusion:**

epidemiological risk factors for COVID-19 and access to healthcare varies between the regions of Cameroon. These discrepancies are potentially reflected in regional differences of COVID-19 cases and case fatality rates. In particular, the East Region has high epidemiological risk factors and low healthcare accessibility compared to other regions. Understanding the relationships between epidemiological risk factors, access to healthcare resources, and COVID-19 cases in Cameroon could aid decision-making among national policymakers and inform further research.

## Introduction

As the pandemic of COVID-19 has spread across the globe, countries have faced diverse challenges. With additional countries facing increasing cases of COVID-19, it is important to realize that experiences and vulnerability may differ among and within countries. In particular, low- and middle-income countries (LMICs) may face particular problems given potential differences in resource availability, demographics, and co-morbidities. An initially lower than expected morbidity and mortality due to COVID-19 has been found in Africa, which some have attributed to a variety of reasons, including a younger population with different co-morbidities and previous exposure to viruses that are cross-reactive with severe acute respiratory syndrome coronavirus 2 (SARS-CoV-2), the virus that causes COVID-19 [1]. Exposure to other pathogens may have also influenced the immune systems of certain African populations, leading to less severity of COVID-19. Recent serological surveys in multiple African countries suggest that large populations may have already been exposed to SARS-CoV-2 or other cross-reactive viruses [2]. However, resource limitations and burden of diseases such as HIV/AIDS, tuberculosis, malnutrition, and malaria could also make certain African populations more vulnerable to COVID-19 [3]. Relatively few assessments of vulnerability for COVID-19 have been undertaken in Africa, and given heterogeneity among African countries, it is crucial to have contextually specific analyses [4]. Additionally, estimates of populations at risk for COVID-19 have been made on national levels in Africa but rarely on regional levels [5]. Limitations in reporting of cases along with co-morbidities may make it challenging to understand specific risk factors and vulnerability. However, existing and emerging epidemiological data may help in guiding these assessments. Vulnerability indices have been used to risk stratify populations based on demographic, health, socioeconomic, and epidemiological variables that could increase vulnerability for COVID-19 [6]. Similar assessments could aid in understanding vulnerability within and between African countries.

Current risk assessments and vulnerability indices for COVID-19 rely on the initial experiences and epidemiological data from primarily Asian, European, and North American countries. These initial epidemiological studies showed a higher proportion of older adults and males among Chinese, Italian, and United States cohorts [7-9]. Hypertension was the most common co-morbidity among the Italian cohort [7]. Diabetes was associated with progression to acute respiratory distress syndrome (ARDS) and death in a Chinese cohort, and it was the most common co-morbidity in a United States cohort [8,10]. Obesity was subsequently associated with increased rate of hospital and ICU admissions among patients in the United States [11]. Malignancy and chronic respiratory disease were also recognized as factors associated with increased risk for ARDS and death in China [10]. Similarly, smoking was also found to be associated with progression of COVID-19 [12]. In a recent cohort study from South Africa, diabetes and hypertension were more common among patients diagnosed with COVID-19 than non-cases, and mortality was associated with increased age, male sex, diabetes, hypertension, and chronic kidney disease [13]. Tuberculosis and HIV were also found to be associated with increased mortality in COVID-19 cases [13]. HIV has not been identified as a risk factor for COVID-19 in previous studies. In a United States cohort, there was no difference in adverse outcomes associated with HIV infection in hospitalized patients with COVID-19 [14]. In Spain, risk for COVID-19 was not higher among HIV positive patients than the general population [15]. Potential confounding variables and differing regional factors such as socioeconomic status, viral load suppression, and access to anti-retroviral therapy make these results difficult to interpret, so more research is necessary. Therefore, previously-identified and presumptive risk factors for COVID-19 could be used for assessing vulnerability in other populations until additional research is available.

While assessing decision-making and outbreak preparedness for emerging viruses in Cameroon, it was found that national policymakers preferred maps and analyses that were region-specific [16]. Cameroon has ten administrative regions and has faced challenges with progression of COVID-19 cases among the different regions [17]. The first cases were detected in the Center Region which contains the capitol, Yaoundé, and subsequently cases spread to the Littoral Region where Douala, the largest city, is located [17]. As of September 16^th^ 2020, 20,371 confirmed cases of COVID-19 had been reported in Cameroon, which ranked ninth among African countries in terms of total cases [18,19]. Cameroon is considered a level 3, high risk country for COVID-19 by the United States Centers for Disease Control and Prevention given ongoing concerns about transmission [20]. In discussions with national experts from Cameroon, it was determined that an assessment of vulnerability for COVID-19 by region in Cameroon could help guide further research. An assessment of differences in epidemiological risk factors for COVID-19 and access to healthcare in Cameroon could help identify particular regions at risk as well as potential mismatches between cases and resource availability. Therefore, we aimed to assess vulnerability for severity of COVID-19 and healthcare access by regions and major cities in Cameroon to identify whether any patterns or discrepancies may exist.

## Methods

To assess regional vulnerability for severity of COVID-19 and healthcare access in Cameroon, two vulnerability indices were developed similarly to those previously described [6,21]. To create an epidemiological vulnerability index, 14 indicators were selected based on prior potential association with increased risk of COVID-19 (Table 1). These indicators were the regional weighted percentage of males or females aged 15-49 who had a history of diabetes/hyperglycemia, heart disease, hypertension, lung disease, smoking, HIV/AIDS, or malignancy. Data for these indicators were obtained from the Cameroon 2018 Demographic Health Survey (DHS) [22]. The DHS separates data from Yaoundé from that of the Center Region, as well as Douala from the Littoral Region because these two major urban cities differ from the other rural areas of the regions. Indicators were min-max scaled from a range of 0-1, with 1 representing higher vulnerability, using the following formula:

X'=(X-min)/(max-min)

X´= scaled value, X= value to be scaled, min = minimum range value of indicator, max= maximum range value of indicator. The epidemiological vulnerability index of each region was then created by summing the re-scaled values for each indicator by region. The same process was used to create the healthcare access vulnerability index. The 3 indicators for the healthcare access vulnerability index included the percentage of females who reported they had difficulty accessing healthcare when they were sick because of distance to a health facility, percentage of females who reported difficulty accessing healthcare when they were sick due to any cause (including difficulty obtaining permission, lacking money for treatment, or not wanting to go alone), and percentage of population in the lowest economic quintile. These indicators were selected because travel time, ability to pay, and economic status strongly influence healthcare access in Cameroon, and there is a very low prevalence of health insurance [23].

**Table 1:** epidemiological vulnerability index for COVID-19 by Cameroon administrative region

	Hypertension		Diabetes		Lung disease		Smoking		Heart disease		Cancer		HIV		Total Index
	F	M	F	M	F	M	F	M	F	M	F	M	F	M	
**Adamawa**	0.16	0.46	0.04	0.11	0.6	0.45	0.22	0.05	0.4	0.21	0	0.5	0.58	0.5	4.3
**Center without Yaoundé**	0.79	0.5	0.21	0.25	0.8	0.62	0.22	0.32	0.37	0.17	0.5	0.17	0.52	0.37	5.8
**Douala**	0.49	1	1	1	0.87	0.6	0.78	0.3	0.75	0.42	0.67	0	0.26	0.2	8.4
**East**	1	0.65	0.43	0	1	0.77	1	1	1	0.20	0	0	1	0.5	8.5
**Far North**	0	0.20	0	0	0.47	0.38	0.11	0.19	0	0.24	0	1	0	0.07	2.7
**Littoral without Douala**	0.45	0.76	0.32	0.32	0.27	0.36	0.11	0.45	0.31	0.18	0	0	0.45	0.07	4.1
**North**	0.21	0.22	0.07	0.14	0.93	0.28	0.22	0.24	0.15	0.13	0.67	0.5	0.23	0	4.0
**North West**	0.15	0.82	0.21	0.18	0.33	0.26	0.11	0.48	0.25	0.05	1	0.5	0.76	0.17	5.3
**South**	0.3	0.26	0.36	0.11	0.07	0.28	0.44	0.24	0.13	0.14	0.5	0.17	0.71	1	4.7
**South West**	0.26	0	0.75	0.36	0	0	0	0	0.15	0	0.17	0	0.42	0.35	2.5
**West**	0.56	0.76	0.79	0.29	0.33	1	0.11	0.14	0.5	1	0.17	1	0.11	0.11	6.9
**Yaoundé**	0.66	1	0.32	0.46	1	0.66	0.33	0.45	0.47	0.38	0.33	0.17	0.44	0.037	6.7

The number of COVID-19 cases, case fatality rates, and associated co-morbidities were gathered from the Cameroon Ministry of Public Health Situation Report from September 16^th^, 2020 [19]. Unlike the DHS, the Situation Report does not separate data from Yaoundé and Douala from their regions. A national survey of the Cameroonian health sector was used for comparison with prior assessments of healthcare capacity by region as well as estimates of population by region and population density [24]. Estimates for percentages of the general population in Cameroon with HIV were obtained from the DHS, and estimates for hypertension and diabetes were calculated using the arithmetic mean from the DHS data. A georeferenced database of public hospitals in Cameroon was used to map and calculate the distribution of general, central, regional, and district hospitals [25]. Maps of regional cases, case fatality rates, hospitals, epidemiological vulnerability indices, and healthcare access vulnerability indices were created using QGIS [26].

## Results

The epidemiological vulnerability index identified the East Region, Douala, West Region, and Yaoundé as being the highest risk for COVID-19 based on percentage of population with potential risk factors for COVID-19 (Table 1). The East Region had the highest weighted percentage of females with hypertension (12.1%), heart disease (3.5%), and HIV (7.3%), as well as males who smoke (19.4%) and females who smoke (0.9%). Douala in the Littoral Region leads among males with hypertension (5.8%) and diabetes (2.8%), as well as females with diabetes (3.2%). The West Region has the highest percentage of males with lung disease (4.7%), heart disease (7.2%), and cancer (0.6%, tied with Far North). Yaoundé in the Center Region leads with percentage of males with hypertension and is tied with the East Region for percentage of females with lung disease (1.8%). The healthcare access vulnerability index showed lower access to healthcare in the North, followed by the Far North, East, and Adamawa Regions (Table 2). The highest percentage of females in the East Region reported difficulty accessing medical care due to any reason (89.8%). Difficulty accessing medical care due to distance was also greatest in the East Region (63.6%). The largest percentage of the population living in the lowest economic quintile were in the Far North Region (52.2%), followed by the North (51.3%) and East Regions (18.7%). The total number of hospitals was greatest in the Center (31), followed by Far North (25), Littoral (22), West (22), North West and South West (18), North (15), East (14), South (12), and Adamawa (7).

**Table 2:** healthcare access vulnerability index for COVID-19 by Cameroon administrative region

	Access to medical care limited by any cause	Access to medical care limited by distance	Population in lowest economic quintile	Total Index
**Adamawa**	0.75	0.94	0.44	2.13
**Center without Yaoundé**	0.85	0.89	0.06	1.8
**Douala**	0.29	0.20	0	0.49
**East**	1	1	0.36	2.36
**Far North**	0.86	0.65	1	2.51
**Littoral without Douala**	0.56	0.57	0.004	1.134
**North**	0.845	0.83	0.98	2.66
**North West**	0.25	0.19	0.26	0.71
**South**	0.62	0.48	0.046	1.14
**South West**	0	0	0	0
**West**	0.43	0.29	0.01	0.73
**Yaoundé**	0.57	0.28	0	0.85

**Figure 1:**
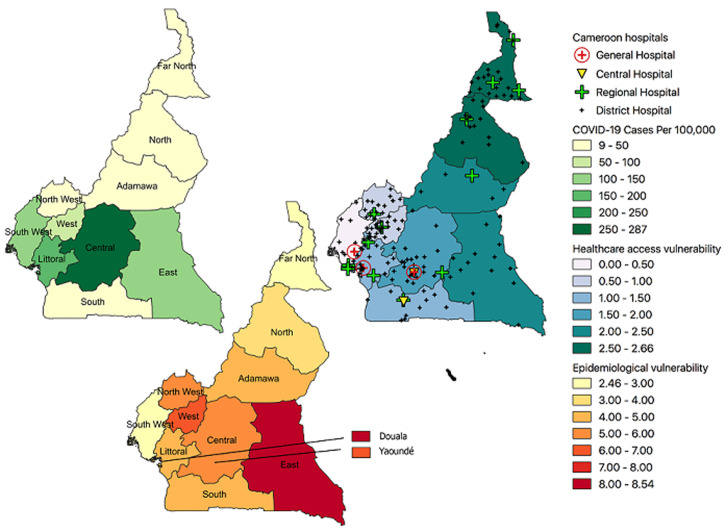
regional vulnerability for COVID-19 in Cameroon

As of September 16^th^ 2020, the distribution of COVID-19 (cases per 100,000 people) in Cameroon by Region have been: Center (287), Littoral (161), East (138), South West (112), West (71.2), North West (46.3), South (45.4), Adamawa (37.5), Far North (13.3), and North (8.9). The case fatality rate of COVID-19 was highest in the North (7.1%), followed by the West (4.7%), North West (4.3%), South West (4.1%), Adamawa (3.4%), East (2.5%), South (2.2%), Littoral (2.2%), Far North (1.9%), and Center (1.1%). The overall case fatality rate for COVID-1 in Cameroon is 2%, and the case fatality rate among HCWs in Cameroon is 2.4%. The regional vulnerability for COVID-19 in Cameroon based on cases per population, case fatality rates, epidemiological vulnerability index, and healthcare access vulnerability index are shown in Figure 1. Among a cohort of hospitalized patients with COVID-19 in Cameroon, co-morbidities included hypertension (21.8%), diabetes (10.7%), HIV (2%), asthma (0.6%), and tuberculosis (1.1%) [19]. Overall, among patients diagnosed with COVID-19 in Cameroon, there were roughly 1.4 times as many males as females, the most prevalent age group infected was 30-39 years (28.3%), and mortality was highest among the 60-69 age group (31.8%). The estimated mean prevalence for diabetes, hypertension, and HIV from the DHS compared to the mean from the hospitalized COVID-19 cohort are shown in Table 3.

**Table 3:** co-morbidities among hospitalized COVID-19 patients vs. general population in Cameroon

	Cohort hospitalized with COVID-19 (%)^1^	Estimated mean population prevalence, ages 15-49 (%)^2^	Estimated mean prevalence, ages 15-49 (%)^2^		Estimated mean prevalence, ages 45-49 (%)^2^	
			F	M	F	M
**Diabetes**	10.7	1.1	1.5	0.75	2.6	3
**Hypertension**	21.8	5.1	6.5	3.7	8	11.9
**HIV**	2	2.7	3.4	1.9	4.9	1.8

Ministère de la Santé Publique. Rapport de situation COVID-19 au Cameroun 16/09/2020. Institut National de la Statistique (INS) et ICF. 2020. Enquête démographique et de santé du Cameroun 2018. Yaoundé, Cameroun et Rockville, Maryland, USA: INS et ICF

## Discussion

From comparing epidemiological and healthcare access vulnerability with cases and case fatality rates of COVID-19 by region in Cameroon, a few observations can be made. In general, COVID-19 cases in Cameroon have centered around the urban areas of each region. The highest number of COVID-19 cases per 100,000 people so far have occurred in the Center and Littoral Regions, which contain the most populous cities, Yaoundé and Douala, that have international airports which likely facilitated the entry of new cases. These regions also have the greatest number of hospitals and relatively better healthcare access than other regions based on the healthcare access vulnerability index. Therefore, the relationship between cases and resource availability appears as one might expect in more densely populated, urban areas. In contrast, the East Region has the lowest population density in Cameroon but the third highest number of COVID-19 cases per 100,000 people. The East Region has the highest epidemiological vulnerability of any region, with a relatively high percentage of the population with HIV, lung disease, hypertension, heart disease, and smoking history. It is possible that these risk factors have contributed to a population in the East Region being more vulnerable to COVID-19. The majority of COVID-19 cases in the East Region have occurred near the city Bertoua, which serves as a major transit point between Yaoundé and Douala with northern Cameroon and other countries such as Chad, the Central African Republic, and the Republic of Congo [27]. It is possible that travel may increase cases of COVID-19 between Bertoua and other areas. Travel patterns have influenced other infectious diseases in Cameroon; for instance, long-distance truck driving in Cameroon has been associated with increased prevalence of HIV/AIDS [28]. The East Region also had the highest percentage of females with difficulty accessing medical care, the third fewest hospitals out of any region, and the third highest healthcare access vulnerability. Therefore, additional healthcare resources may be needed in the East Region to correspond with the proportion of COVID-19 cases and population vulnerability.

Along with the East, the North, Far North, and Adamawa Regions have the highest healthcare access vulnerability indices. These regions also have the highest population percentages living in the lowest economic quintile [22]. From a survey of the Cameroonian health sector in 2009, regions were grouped into better-served and underserved based on concentration of healthcare facilities compared to population density [24]. The most underserved regions were the East, Adamawa, North, and South, and these regions also ranked high on the healthcare access vulnerability index. Rural populations with less healthcare resources often rely on traditional medicine in Cameroon [29], and individuals from these areas may not present to hospitals to be diagnosed or treated for COVID-19. Therefore, regions with high epidemiological and healthcare access vulnerability may be at increased risk for COVID-19, and it will be important to identify any mismatches between cases and resources. Regions in Cameroon with relatively large numbers of COVID-19 patients will not only require additional resources such as hospital beds, medications, and ventilators, but also will need personal protective equipment (PPE) for HCWs. Clinicians and patients have raised concerns about the lack of PPE and resources available at hospitals in Cameroon during the COVID-19 pandemic [30]. As of September 16^th^ 2020, 836 HCWs in Cameroon have been diagnosed with COVID-19 [19], and more HCWs are at risk for infection due to the propensity of SARS-CoV-2 for nosocomial transmission [31]. Furthermore, diagnostic capacity for COVID-19 varies by region. There are currently 15 operational diagnostic laboratories for COVID-19 in nine of ten regions, with only the South Region lacking a laboratory with polymerase chain reaction (PCR) capacity [19]. Given differences in travel times to hospitals and availability of diagnostic testing between regions, it is possible that certain regions may have relatively more undetected cases or delays in diagnosis, which could lead to more local transmission. Comparing emerging data from the Cameroon Ministry of Public Health Situation Report with prior population data from the DHS reveals a few patterns about potential risk factors for COVID-19 in Cameroon. Hypertension and diabetes were the most prevalent co-morbidities among a cohort of patients hospitalized with COVID-19 [19]. Diabetes was roughly ten times more prevalent among those with COVID-19 compared to the estimated mean in the national population and hypertension was approximately four times more prevalent. HIV was not more prevalent among the COVID-19 cohort than the estimated population mean. More males were diagnosed with COVID-19 than females. The higher prevalence of COVID-19 among males and those with diabetes and hypertension indicate that these may be possible risk factors in Cameroon. However, additional data and case control studies are necessary for further understanding particular risk factors.

There are multiple limitations to this analysis. Data on the prevalence of co-morbidities in the elderly, obesity, and chronic kidney disease in Cameroon were not available, which are recognized as epidemiological risk factors in other countries. Given uncertainty regarding the relative contribution of different risk factors for COVID-19, indicators were weighted evenly but likely vary in significance. Additionally, vulnerability assessments of the South West Region are likely biased and underestimated given that data from that region reflected mainly the urban areas due to security issues during data collection as a result of the ongoing civil war. While other vulnerability indices for COVID-19 have included additional demographic data such as population density and hygiene [6,21,32], the focus of our study was on epidemiological risk factors and healthcare access and did not include these data. Hospital data were also primarily focused on the public healthcare system and therefore may miss certain hospitals in Cameroon. This analysis also does not take into account that people may move between regions for healthcare, generally from rural to urban areas. Data on difficulty accessing medical care among males were not available, and males may be at greater risk for COVID-19. The ages of the hospitalized COVID-19 cohort with co-morbidities are not available, but presumably they may represent an older population than the 15-49 age group in the DHS from which mean population estimates of co-morbidities were made. The weighted percentages of populations with co-morbidities from the DHS also relies on availability of diagnosis by healthcare providers, which may vary by region. Likewise, diagnostic testing for COVID-19 also varies by region, which may lead to an underestimate of cases and case fatality rates in certain regions. In particular there may be undiagnosed and unreported cases or deaths in certain communities. These limitations indicate the importance for further research. For instance, randomized serological surveys could be used to provide estimates of COVID-19 prevalence between the regions of Cameroon.

## Conclusion

Despite multiple limitations, comparing vulnerability indices and co-morbidities with emerging data may be helpful in identifying regional differences in risk factors for COVID-19 in Cameroon and other LMICs. For example, the East Region has a high number of cases and potential co-morbidities per population but lower access to healthcare. Overall, additional data are needed for further analysis, but our results could help researchers understand which regional populations may have particular risk factors for COVID-19. National researchers in Cameroon plan to use our epidemiological vulnerability index to identify which populations to study for host and viral determinants of COVID-19 severity. Comparing emerging data on COVID-19 cases with estimates of population co-morbidities reveal that male sex, diabetes, and hypertension may be risk factors for COVID-19 in Cameroon. Additional reporting of co-morbidities in patients with COVID-19 could help public health experts further determine risk factors for populations in Cameroon and other African countries. Along with the healthcare access vulnerability index, additional analyses on resource availability such as hospital beds, ventilators, and PPE could help policymakers decide where to allocate resources in preparation for a surge in cases. As African countries continue to face cases of COVID-19, it will be important to conduct regional analyses to examine patterns of risk factors and healthcare access. Vulnerability for COVID-19 differs within and between countries, and both international and local partnerships between HCWs, public health experts, and scientists will be necessary to understand these differences.

### What is known about this topic

- Epidemiological studies have identified risk factors for COVID-19 but are primarily based on patient cohorts from the Asia, Europe, and North America; there are limited studies on co-morbidities and risk in Africa;
- Vulnerability indices based on potential epidemiological risk factors and healthcare resources may be helpful in identifying populations at risk for COVID-19;
- National policymakers in Cameroon value region-specific maps and analyses for decision making regarding outbreak preparedness.

### What this study adds

- Epidemiological vulnerability for COVID-19 varies by region in Cameroon given particular distributions of potential co-morbidities;
- Healthcare access varies by region in Cameroon, leading to potential discrepancies in diagnosis and treatment for COVID-19;
- Identifying regions that have high epidemiological vulnerability and low access to healthcare may help identify resource mismatches and populations at increased risk for COVID-19 in Cameroon and other African countries.
